# Digital Media, the Right to an Open Future, and Children 0–5

**DOI:** 10.3389/fpsyg.2018.02137

**Published:** 2018-11-06

**Authors:** Monika Sziron, Elisabeth Hildt

**Affiliations:** Center for the Study of Ethics in the Professions, Illinois Institute of Technology, Chicago, IL, United States

**Keywords:** digital media, child-computer interaction, ethics, autonomy, privacy, digital divide

Many parents share the lives of their children from the ages of 0–5 via digital media. Children are even publicized before birth via ultrasound images (Taylor and Rooney, 2017). Sharing a child's life with the public is not novel. Parents have been sharing the lives of their children for centuries via photographs and photo albums. However, the debate is still ongoing as to the implications of whether sharing a child's life via digital media is comparable, or incomparable, to these previous analog methods (Brosch, 2016). In addition, there is now the awareness that children 0–5 are increasingly users of digital media themselves, which may lead to creating a digital footprint without knowing it (Nansen, 2015). A limited amount of data exists on internet and digital media use by children ages 0–5 (Miller et al., 2017).

According to the UK Children Commissioner's report “Growing Up Digital” (Children's Commissioner, 2017), children 3–4 spend, on average, 8 h a week online (in 2017), having grown considerably in the preceding 12 months. In view of the substantial amount of time young children are spending with digital media, there is ongoing discussion of what adequate technology use for children 0–5 looks like, with regard to the modality and the time spent, so that it is beneficial for young children and not negatively affecting them. The American Academy of Pediatrics recommends limiting the duration of total entertainment screen time for children to no more than 1 h a day and discourages screen media exposure for children under 2 (Reid Chassiakos et al., 2016). Others have criticized screen time restrictions like these as not realistic (Nansen, 2015). Despite where one's position is in this debate, it is obvious that there is a need to develop an adequate framework for digital media use and investigate thoroughly the benefits, risks, and implications of digital media use in very young children. Furthermore, increased awareness is needed for the role adults—parents, caregivers, and the like—play for digital media use in young children and for children's digital footprints. With this article, we would like to contribute to this development, focusing on the social and ethical implications of digital media technology relating to children 0–5.

One way for adults to approach the debate, from a philosophical-ethical perspective, is by referring to the concept of a Child's Right to an Open Future. The right to an open future coined by Feinberg (1980), encompasses moral rights to be saved for the child until the child is an adult. For children are not yet autonomous but will one day become capable of autonomous decision-making. According to Feinberg, the “child's right to an open future” is a set of autonomy rights-in-trust. Children cannot exert these rights yet, but these rights can be violated before the child acquires the capability to act autonomously. The child's right to an open future is the right to have future options open until they reach adulthood. While it may be questioned whether digital technology use will ever be so influential to significantly violate a child's right to an open future (Millum, 2014), several arguments do center around positive or negative influences on a child's future options.

On a moderate interpretation, the child's right to an open future can be seen as the child's right to acquire a reasonably broad spectrum of capacities, skills, and opportunities (Millum, 2014). The central question here is whether and how digital technologies positively or negatively influence children's development, abilities, knowledge, and emotional and social skills. Similarly, an approach based in capability theory focuses on the extent to which digital technologies increase the capability of children (as future adults) to shape and realize lives that they value (Johnstone, 2007). Capability-based questions are whether digital media technologies increase children's communication, knowledge, or abilities; whether technology use reduces opportunities for interpersonal communication, social and emotional experiences, or outdoor activities; or whether the prevalence of digital technologies causes a reduction of the cultural richness of children's environments (Johnstone, 2007).

The history of children, rights, and citizenship has changed throughout the years. Thanks to the Geneva Declaration of the Rights of the Child (1924), the Declaration of the Rights of the Child (1959), and the Convention of the Right of the Child (1989), children are subjects of rights. In the age of digital media, children are increasingly participating in digital environments in which they are considered to be digital citizens. As presumed digital citizens, they are expected to act ethically, responsibly, and knowledgeably when using digital media, even though young children clearly have limited capacity to do so.

As the discussion of children and citizenship posits many questions, so does the discussion of children and digital citizenship. Does digital citizenship pertain to children ages 0–5? At what age should a child learn how to be a digital citizen? Who is responsible for ensuring that children are good digital citizens? For example, the UK Children Commissioner's report (2017) suggests a digital citizenship program, compulsory in every school for children ages 4–14. We advocate that digital citizenship needs to include culturally responsible use of technology that ideally translates into physical interactions as well. When we use the term ‘culturally responsible’ we want to highlight the use of digital media to respect diversity and to not discriminate, bully, or harass others. Culturally responsible use of technology also includes maintaining and respecting not only others' privacy, but one's own privacy.

Young children, ages 0–5, are not able to make autonomous decisions, and lack awareness of and ability to control possible negative consequences of their online activity and privacy. There is consent that special protections for children online have to be in place, as expressed in the OECD Recommendation on the Protection of Children Online (OECD, 2012). The European Union's General Data Protection Regulation (European Union, 2016; Macenaite, 2017) describes specific measures to protect children's personal online data. Among children online, privacy protection measures require parental consent before children's personal data is processed, and the right to be forgotten allows children to eliminate personal data. Further measures are the provision of “data protection by design” standards, and the prohibition of profiling. In this context Milda Macenaite discusses the “empowerment vs. protection dilemma,” the dilemma of finding a balance between offering choices and empowering children to use the online opportunities available for playing, communicating, and learning while also protecting them (Macenaite, 2017).

However, it has to be seen that the younger the child, the higher the degree of protection required, insofar there is not much that can be brought forward against high protection for very young internet users 0–5. This is especially true for general data protection that requires high level privacy settings to be installed by default. Parents giving proxy consent for their young children in a considerate way is an effective measure to protect their children's privacy, and teaches them to gradually get used to the digital world and assume responsibility for their personal information. The right to be forgotten provides a path to delete digital information that may have been created unnecessarily or that may no longer be needed, reducing a child's and future adult's digital footprint.

Justice requires that benefits related to digital media technologies should be distributed equally. However, several factors lead to inequalities and digital divide. Socioeconomic status plays a considerable role, as technological devices and internet access require financial resources (Miller et al., 2017). We also want to address the facets of the digital divide in terms of access to active vs. passive technologies and the divide between media literacy and media use. It is not just about access to digital media technologies, children need access to digital media technologies that permit active participation. This includes educational games and interactive media, rather than media that only require passive viewing or very little interaction. The divide between active and passive technologies can be correlated with the divide between media literacy and media use. When a child has to actively engage with digital media, they are more likely to learn media literacy (Kirkorian, 2018). When a child is passive in their digital media consumption, this will often reflect little media literacy and lead simply to media use (Office of Educational Technology, 2018). This becomes a concern as research has found that children who engage with digital learning resources are better prepared for Kindergarten than children who do not have access or have limited access (Lozano et al., 2016). Yet, varying levels of interactivity are necessary, “Not all types of interactivity increase learning and not all children learn to the same degree” (Kirkorian, 2018, p. 212). Distinguishing the needs of children ages 0–5 is crucial for learning how to best support them.

There are manifold ways for children 0–5 to learn digital technology use, such as through demonstration, guided interaction, copying, dialogue, family practices, and sociocultural embeddedness, even though it has been suggested that parents tend to underestimate their role (Plowman et al., 2008; Nansen, 2015). A video study by Danby et al. (2013) illustrates complex verbal and non-verbal interaction between a father and two young children using digital devices. The authors characterize the interaction between family members as “talk in activity,” i.e., engaging in social interaction and conversation while using digital devices.

When children use digital media there is also a risk of trying to “be like adults.” It can be argued that children ages 0–5 are always learning from adults and trying to be like the adults around them. However, with the use of digital media, children are increasingly engaging in environments that are designed by and for adults. We posit that children may benefit from their own digital media environments like that of Facebook's Messenger Kids. However, digital environments of this kind still give rise to the potential of children being the targets of adult-like marketing. Children ages 0–5 have been marketed as mini-adults rather than children. Digital media inspired toys, games, and online behavior often reflect adult needs, rather than the needs of children. For example, a toy for ages 18 months and up, is promoted as “Your budding techie can act like a grown up with this Kidz Delight Tech Too & Phone Set.” A toy, for ages 2–5, is marketed as “Play and talk just like mom and dad on your very first smart phone,” and “The Tech Too Tech Set includes kid friendly versions of three of the most popular and iconic grown-up devices…” Researchers and developers should continue processes like Cooperative Inquiry that involve children in the design process of these kinds of technologies (Guha et al., 2013). Further research should specifically focus on how children ages 0–5 can take part in Cooperative Inquiry. Fails et al. (2013) provide methods and techniques for developing new technologies with and for children, rather than without children and by adults.

The implications of digital media use have consequences, both positive and negative, on a child's development. Understanding these consequences is important for parents, but also engineers and developers working in child-computer interaction. While studies may describe situations in which comprehensive support by parents or educators is provided, it may be questioned whether in daily family routine, children always have adequate support available that enables them to have deep learning experiences or comprehensively positive experiences while using digital devices. There is a clear need to actively accompany very young children's digital media use in order to avoid time spent with non-inspiring and mere repetitious passive consumption of media, but also to provide an adequate framework guaranteeing data protection and privacy.

A child's right to an open future requires parents, software engineers, designers, policy makers, and marketers understanding the ramifications of digital media for ages 0–5. Children's first interactions with digital media need to be supplemental for developing autonomy, digital citizenship, and culturally responsible use of technology, while avoiding negative privacy implications, the risks of digital divide, and “being like adults.” Ethical development of future digital media technology should take into consideration the child, as a child with an open future, in the hopes of maintaining that open future.

## Author contributions

All authors listed have made a substantial, direct and intellectual contribution to the work, and approved it for publication.

### Conflict of interest statement

The authors declare that the research was conducted in the absence of any commercial or financial relationships that could be construed as a potential conflict of interest. The handling Editor declared a shared affiliation, though no other collaboration with the authors.
